# Beyond the silos: A global evidence synthesis of biodiversity, water, food, health and climate nexus interactions

**DOI:** 10.1016/j.isci.2026.116871

**Published:** 2026-07-22

**Authors:** Paula R. Prist, Allison Bailey, Elena Bukvareva, Eva Maire, Ralf Seppelt, David T.S. Hayman, Lisa Biber-Freudenberger, Sunita Chaudhary, Pradeep Kumar Dubey, Ronald C. Estoque, Yuka Estrada, Gábor Földvári, Paula A. Harrison, Abid Hussain, Tiff van Huysen, Roxanne Suzette Lorilla, Silvia Francis Materu, Pamela McElwee, Ernest L. Molua, David Obura, Giles B. Sioen, Caroline Howe

**Affiliations:** 1 IUCN, Forest and Grasslands Programme, 1630 Connecticut av. Ste 300, Washington, DC 20009, USA; 2 Department of Biological Sciences, University of Notre Dame, Notre Dame, IN 46556, USA; 3 Biodiversity Conservation Center, BCC-Armenia, Jrvezh 2224, Armenia; 4 MARBEC, University of Montpellier, CNRS, Ifremer, IRD, Montpellier, France; 5 Lancaster Environment Centre, Lancaster University, Lancaster LA1 4YQ, UK; 6 Luxembourg Centre for Socio--Environmental Systems (LCSES), Luxembourg University, Esch-- sur-- Alzette, Luxembourg; 7 Molecular Epidemiology and Public Health Laboratory, School of Veterinary Sciences, Massey University, Palmerston North 4442, New Zealand; 8 Center for Development Research, Bonn University, Genscherallee 3, 53113 Bonn, Germany; 9 International Centre for Integrated Mountain Development (ICIMOD), Kathmandu, Nepal; 10 Department of Agronomy, Institute of Agricultural Sciences Banaras Hindu University (BHU), Varanasi, Uttar Pradesh 221005, India; 11 Center for Biodiversity and Climate Change, Forestry and Forest Products Research Institute, Tsukuba 305-8687, Japan; 12 Independent Researcher, San Francisco, CA, USA; 13 Institute of Evolution, HUN-REN Centre for Ecological Research, Budapest, Hungary; 14 Centre for Eco-Epidemiology, National Laboratory for Health Security, Budapest, Hungary; 15 UK Centre for Ecology & Hydrology, Lancaster LA1 4AP, UK; 16 International Centre for Integrated Mountain Development, Kathmandu, Nepal; 17 Intergovernmental Science-Policy Platform on Biodiversity and Ecosystem Services (IPBES), 53113 Bonn, Germany; 18 Department of Geography, Harokopio University of Athens, El. Venizelou 70 Str, Kallithea, 17676 Athens, Greece; 19 Department of Biosciences, College of Natural and Applied Sciences, Sokoine University of Agriculture, P O Box 3038, Morogoro, Tanzania; 20 Department of Human Ecology at Rutgers, The State University of New Jersey, New Brunswick, NJ 08901, USA; 21 Department of Agricultural Economics and Agribusiness Faculty of Agriculture & Veterinary Medicine, University of Buea, Buea, Cameroon; 22 CORDIO East Africa, Mombasa, Kenya; 23 Sustainable Society Design Center, Graduate School of Frontier Sciences, The University of Tokyo, Kashiwa, Chiba, Japan; 24 Centre for Environmental Policy, Imperial College London, South Kensington, London SW7 2AZ, UK

**Keywords:** nexus, sustainability, conservation, interactions, IPBES, feedback loops, complexity, biodiversity

## Abstract

Healthy, resilient ecosystems depend on biodiversity to provide ecosystem services that are essential for human well-being. However, unsustainable human activities have driven biodiversity loss, ecosystem degradation, and climate change, generating cascading impacts on water, food, and health systems. As part of the Intergovernmental Science-Policy Platform on Biodiversity and Ecosystem Services (IPBES) Nexus Assessment, we reviewed 1,962 articles and identified 84 that explicitly addressed biodiversity and at least three other nexus elements. Across these studies, we documented 477 interactions, including 188 positive synergies, 148 negative synergies, and 141 trade-offs. Human health was the least represented nexus element and was largely treated as an outcome rather than a driver. Biodiversity emerged as foundational to positive synergies across the nexus, while climate change drove mutual losses and food production generated multiple trade-offs. These findings highlight the need for integrated policies that simultaneously conserve biodiversity, transform food systems, and address climate change to generate co-benefits for water, food, and human health.

## Graphical abstract

**Figure:**
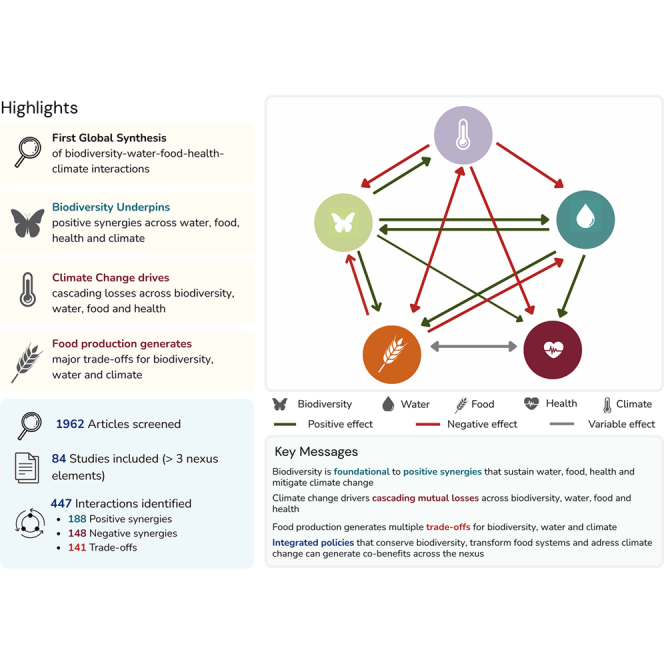

## Teaser

Global change; Nature conservation; Ecology

## Introduction

The interconnections between biodiversity, water, food, health, and climate change are profound, and the degradation of one element can affect other elements in a complex web of interrelated, compounding and cascading risks.^1^^,^^2^ Over the past 70 years, people have transformed natural environments more rapidly and extensively than in any comparable period in history,^3^^,^^4^^,^^5^ and today almost all ecosystems are under pressure from anthropogenic and climate change impacts, contributing to the transgression of six of the nine planetary boundaries.^6^ Cropland and pastures have expanded by 1.9 million km^2^ between 1960 and 2019. The area of primary forest worldwide has decreased by over 80 million hectares since 1990,^7^^,^^8^ and approximately 25–50% of the past extent of coastal wetlands (including mangroves, tidal marshes and seagrasses) have been lost in the past 50 years.^9^^,^^10^ Consequently, 30% of the land area can be considered currently highly or moderately transformed,^11^ 59% of the ocean is experiencing significant increasing cumulative impact,^12^ and biodiversity is rapidly declining across the globe. Since 1500, about 30% (uncertainty range: 16–50%) of species have become globally threatened or driven to extinction,^13^ with variations across major taxonomic groups from 12% for birds to 71% for cycads.^14^ Populations inhabiting human-modified landscapes are losing their within-population genetic diversity,^15^ and a homogenization of biodiversity has been observed at larger scales.^16^^,^^17^^,^^18^ Such losses are expected to have far-reaching consequences, given that well-functioning, stable and resilient ecosystems fundamentally depend on genetic and species diversity to provide essential goods and services, as well as physical, spiritual, and mental benefits vital for human health and well-being.^19^^,^^20^^,^^21^^,^^22^^,^^23^^,^^24^^,^^25^^,^^26^

Healthy terrestrial ecosystems, particularly forests, regulate all stages of the hydrological cycle, providing clean water for people.^27^^,^^28^ Biodiversity plays a central role in all food systems and contributes to food and nutritional security.^29^ Biodiversity also directly and indirectly contributes to human health, from our microbiomes to providing resources for traditional and modern medicine, as well as being essential to mental health^30^ and controlling transmission risks of some pathogens through the dilution effect.^31^ There is also a bidirectional interconnection between biodiversity and the ecosystem regulation of climate,^32^ with even small changes in climatic conditions triggering ecological systems to alternative states which can be hard to reverse.^33^

Nexus studies have become increasingly important in outlining the interconnections, complexities, and feedbacks among different elements. However, the majority of existing studies assessed historical trends in biodiversity, water, food, health, and climate change individually, or in bilateral interactions, ignoring the complexity and interconnections between these different elements.^34^^,^^35^^,^^36^^,^^37^^,^^38^^,^^39^^,^^40^^,^^41^^,^^42^ Nexus thinking emerged as a response to such fragmentation by providing a holistic approach for understanding how interconnected systems interact across sectors, scales, and governance domains, and for identifying solutions that maximize synergies while minimizing unintended trade-offs.^43^^,^^44^

While the water-energy-food (WEF) nexus has become one of the most widely applied nexus frameworks, it represents only one expression of the broader systems perspective underpinning nexus thinking.^44^^,^^45^ As the field has evolved, nexus approaches have expanded beyond the original WEF configuration to incorporate additional social, ecological, and economic dimensions. This has occurred both through the addition of new components, such as climate, biodiversity, or soil, and through the development of alternative nexus configurations that reflect different sustainability challenges and system relationships, such as the Biota-Food-Energy-Climate (BFEC) nexus.^45^^,^^46^ Despite these advances, biodiversity and health have remained underrepresented, being frequently treated as environmental and social outcomes, respectively, rather than as foundational components that actively shape system dynamics and influence interactions across the nexus.

The Thematic Assessment Report on the Interlinkages among Biodiversity, Water, Food and Health of the Intergovernmental Science-Policy Platform on Biodiversity and Ecosystem Services (IPBES) (or “Nexus Assessment”) applies a nexus thinking to understand the interconnections between biodiversity-water-food-health and climate.^47^ By explicitly recognizing biodiversity and health as foundational, co-equal components of the system, it enables a more comprehensive understanding of the reciprocal relationships linking ecological integrity, human well-being, and sustainable development. Other dimensions are incorporated within this framework: Sanitation (including WASH) is embedded within the water and health dimensions as a fundamental service delivery mechanism; land and ecosystems form the spatial and ecological foundation of the nexus, with ecosystem being captured through biodiversity and its functions, and land being addressed primarily within the biodiversity and food dimensions. Other sectors, such as energy, infrastructure, or governance, are acknowledged as important enabling conditions that influence long-term sustainability. However, they fall outside the primary objectives of this study and are therefore considered as contextual factors rather than core nexus components. In addition, by including five elements, it also expands the interactions, feedback loops, and potential leverage points, revealing dynamics that may remain hidden in narrower configurations.^46^

Here, we perform a synthesis of the scientific literature to assess the status and trends of interactions between biodiversity, water, food, health and climate change (henceforth referred to as the nexus). By elucidating these interdependencies and assessing the complexity of this nexus, this article provides evidence to better understand and address the multifaceted nature of global change. This is particularly important as the escalation of any single crisis–such as biodiversity loss, climate change, food and water insecurity, or pandemics–can amplify others, reinforcing systemic vulnerabilities to global change. Understanding these interactions is a precursor not only to developing better responses but also to providing proactive policy recommendations to tackle this polycrsis.

### Systematic review

Data on the interactions between biodiversity, water, food, health and climate change were assessed through a systematic literature review using the *litsearchr* R package (version 1.0.0), a tool that partially automates search term selection and produces search strategies for systematic reviews. *Litsearchr* uses the Rapid Automatic Keyword Extraction algorithm^48^ to identify potential keywords from a sample of titles and abstracts and combines them with database-tagged keywords to create a pool of possible keywords relevant to a field of study.

With the aim of capturing the most important search terms grouped into concept topics or categories of synonymous terms,^48^ a set of naive keywords was chosen and used in Scopus on 21 July 2022 without year limitations, which returned 686 articles (see Table 1 for the naive and final keywords used). The search happened in 2022 to align with the timing of the Nexus assessment work. Those 686 articles were exported into an EndNote library and imported to R, serving as input for the *litsearchr* package (version 1.0.0). Duplicates were removed, and the *extract-terms* function was utilized to systematically extract all potential keywords from the article titles, keywords, and abstracts. A keyword co-occurrence network was created to measure the importance and influence of each term in relation to the topic being reviewed. To find the best terms, the number of times that each term was used in titles, abstracts, and keywords was ranked from the highest to the lowest number and assigned as a strength value for each term.

**Table 1:** Naive and final keyword combinations used in the systematic review about the interactions between biodiversity, water, food, health, and climate change

Naive keywords	Final keywords—suggested by *litsearchr*
(Biodiversity OR diversity∗ OR landscape∗ OR ecosystem) AND (Food OR food∗ OR food security OR productivity OR nutrition) AND (Water OR water∗ OR hydro∗) AND (Climate OR Climate change OR Climate∗) AND (Human health OR well-being OR human∗ OR zoono∗)	(“ecosystem service” OR “natural resource” OR “sustainable development” OR “environmental sustainability” OR “natural ecosystem” OR biodiversity) AND (“human health” OR “human well-being” OR zoonotic OR “mental health”) AND (“water management” OR “water quality” OR “water security” OR “water use” OR “water availability” OR “water resource” OR “water supply”) AND (“crop yield” OR “food production” OR “food security” OR “agricultural production” OR “production system” OR nutrition) AND (“carbon sequestration” OR “climate change” OR “greenhouse gas”) AND (“environmental change” OR “environmental impact” OR “human activities” OR “land degradation” OR “land management” OR “land use” OR “economic development” OR “human population” OR “population growth”).

The terms that appeared in at least 3% (*n* = 20) of the articles were selected as final keywords. This threshold was chosen after testing the values of 1, 3, 5, and 10% to see which captured the highest number of terms that were significant for the study. As there was no significant difference between those values and to capture the largest number of terms, we opted to use the 3% threshold. After expert review by the author team, additional highly relevant keywords were also included to ensure the search would capture studies involving biodiversity, water, food, health and climate change and their interactions (Table 1). With this combination, a new *Scopus* search was performed on 22 July 2022 using the different nexus interactions keywords. The number of articles found within each nexus combination, using the keywords provided by the *litsearchr* package, can be found in Table S1.

**Figure 1:**
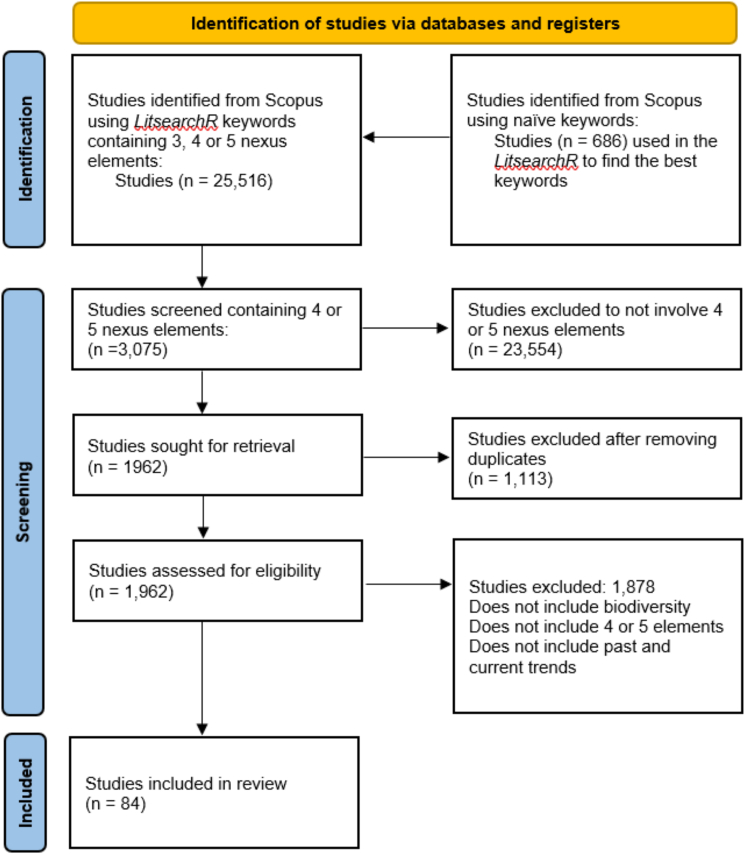
PRISMA flow diagram for the systematic review process carried out in 2022 to align with the timing of the production of the IPBES Nexus Assessment (and without year limitation), to extract data on the interactions between biodiversity, water, food, health and climate change, starting with the use of naive keywords for keyword selection through the *litsearchr* package in R

The sum of the searches involving all possible combinations of the nexus elements produced 25,516 articles without considering duplicates (see Table S1 for more details). However, as our goal was to capture interactions among at least four elements, this reduced the number of articles to 3,075, which, after removal of duplicates, resulted in 1,962 articles (Figure 1).

These 1,962 articles were read in their entirety, and only articles that contained empirical evidence and biodiversity as one of the elements of the nexus being studied together with three or more other elements were included for data extraction. Biodiversity was an inclusion criterion since it is considered a foundational element within the IPBES Nexus Assessment. Following a full-text screening by at least three authors, a total of 84 articles were retained, with 477 interactions coded, as most articles contained more than one interaction. All peer-reviewed articles written in English were considered (acknowledging the potential bias from only considering English-language articles^49^). The systematic review followed the PRISMA guidelines.^50^

The goal of the coding process was to extract how one nexus element (“entry point”) could affect the other elements and in what direction. As such, the geographic location (country, multiple countries or global), the system (e.g., marine, forest), the nexus elements involved in the study, the trend of the entry point, the direction of effect on the other nexus elements, the indirect and direct drivers, and the indicators used for each element were extracted. Direct and indirect drivers were classified according to the IPBES Global Assessment.^19^ Direct drivers are the proximate human-caused pressures that directly impact biodiversity, including land/sea use change, unsustainable exploitation of resources, climate change, pollution, and invasive alien species. These direct drivers are underpinned by economic, demographic, cultural, institutional and technological indirect drivers.^19^^,^^51^ For clarity, and because each element of the nexus was measured in different ways, interactions between the elements were grouped according to summarized categories or indicators used in the 84 articles (Table 2).

**Table 2:** Summarized categories or indicators with descriptions, used in the studies as components of the nexus elements of biodiversity, water, food, health, and climate change

Element of the nexus	Category/indicator	Description
Biodiversity	wild species	presence and propagation of wild animals
	cultivated species	plant species used in agriculture or ranching practices
	ecosystems	status, quality, or composition of terrestrial or aquatic ecosystems
	biodiversity (general)	articles not specific about biodiversity definition
Water	water availability	presence or accessibility of surface, glacier, or groundwater
	water movement	abnormal water movement and interaction with environment (e.g., dams, irrigation, flooding)
	water quality	chemical or physical state/condition of water
Food	consumption and diets	food processing and variety consumed across global environments
	emissions and pollutants	toxins from agricultural processes affecting ground, water, or atmosphere negatively
	production	methods/processes used in agriculture or ranching to produce food, fiber, or commodities
	resources needed	inputs required to support food production systems such as cattle feed or pastures
Health	communicable diseases	illness caused by pathogens, such as bacteria, viruses, fungi, parasites, that can spread from one person or animal to another, or from a contaminated environment, i.e., infectious diseases.
	non-communicable diseases	chronic non-infectious diseases not passed between people, e.g., diabetes, heart disease
	human mortality	death rates attributable to various factors, including climate change impacts
	mental and physical health	general human wellness, including mental health
Climate change	climate change and drivers	general phenomenon of climate change and drivers like greenhouse gases or carbon sequestration
	extreme events	flooding, drought, and other extreme weather events
	temperature and precipitation changes	changes in climatic temperature and rainfall patterns

The data were organized to track how trends in one nexus element (i.e., entry point) affected the other elements. To help with interpretation, for climate change we coded positive trends to indicate climate change mitigation (i.e., improvements), while a negative trend indicates intensifying climate change. For the other elements, increases indicate positive impacts on biodiversity, water, food and health, while decreases indicate degradation of those elements. Relationships were classified as positive synergies, here called mutual gains, negative synergies, or mutual losses, and trade-offs, for those with opposing or variable outcomes (i.e., enhancement or desirable outcome in one element leading to a decrease or deterioration in another element).

To quantify the overall effect between two nexus elements, we calculated a relative weight by summing across all relationships found. For example, if 13 studies found that increases in biodiversity lead to positive effects on water availability, while four studies found the opposite (negative effects), the overall effect for the trend “biodiversity increase on water” received a weight of 9 (13–4). Similarly, if four studies reported that decreases in biodiversity were positive for water quality and two found contrasting results, the overall effect for the trend “biodiversity decrease on water” was assigned a weight of 2 (4–2). Finally, the relative weight for the general effect of “biodiversity and water” was calculated by combining the weights of all relevant indicators, e.g., 11 [(13–4) + (4–2)] in this example. This process was applied for all interactions. To summarize the overall effect of observed positive and negative synergies and variable effects (i.e., trade-offs), we calculated relative weights by separating the data into positive and negative trends of the input element (i.e., increases in biodiversity, water, food, and so forth) following the same logic described above. In this step, the number of studies was also used to determine the level of evidence, classified into four categories: 1 to 9 studies as inconclusive evidence; 10 to 19 as unresolved evidence; 20 to 29 as established but incomplete evidence; and 30 to 40 as well-established evidence (using IPBES terminology for assessing the degree of confidence^52^). When the number of studies supporting different effects was the same, the effect was classified as neutral. If no studies addressed a given interaction, it was assigned “no evidence.”

## Results

### General patterns and knowledge gaps

**Figure 2:**
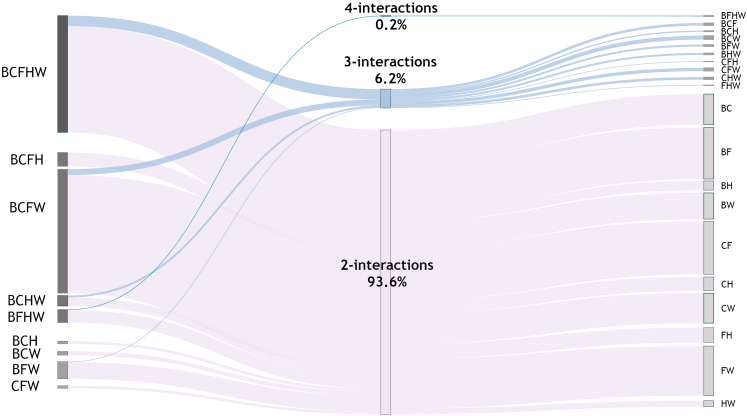
Differences in interactions among the nexus elements addressed (left column) versus nexus interactions analyzed (right column) in articles from the literature review of multi-element interaction studies Most interactions studied in the 84 articles selected evaluated relationships between two elements of the nexus only (in 93.6% of the cases), with a few considering three (6.2%) and only 1 study (0.2%) considering interactions among four elements of the nexus (middle column). Capital letters represent the five nexus elements: B: biodiversity, C: climate change, F: food, H: health and W: water. Four-nexus element interactions are represented by light green color, three-interactions by blue and two-interactions by light pink.

The literature review resulted in 84 relevant publications analyzing four or five nexus elements, published between 1997 and 2022, given a literature cut-off date of November 2023 for inclusion in the assessment. Even though the 84 articles mention at least four- and five-way interactions, after the analysis and coding extraction process, most studies were found to have evaluated interactions through cause-and-effect relationships between only two (in 93.6%) or, at most, three nexus elements (in 6.2%) (see Figure 2). Thus, although biodiversity is included in all articles, it is not included in all interactions found. For example, in Hoy et al. (2016),^53^ the goal of the article was to evaluate the effects of climate change on water, biodiversity, food, and human health. However, the effects of climate change were assessed through direct relationships with each element of the nexus without assessing their cascading effects on the other nexus elements, i.e., the increase in the effects of temperature on the phenology, composition, and distribution of forest species; the increase in temperature affecting crop yields; and the increase in temperature leading to an increase in the spread of vector-borne diseases.

**Figure 3:**
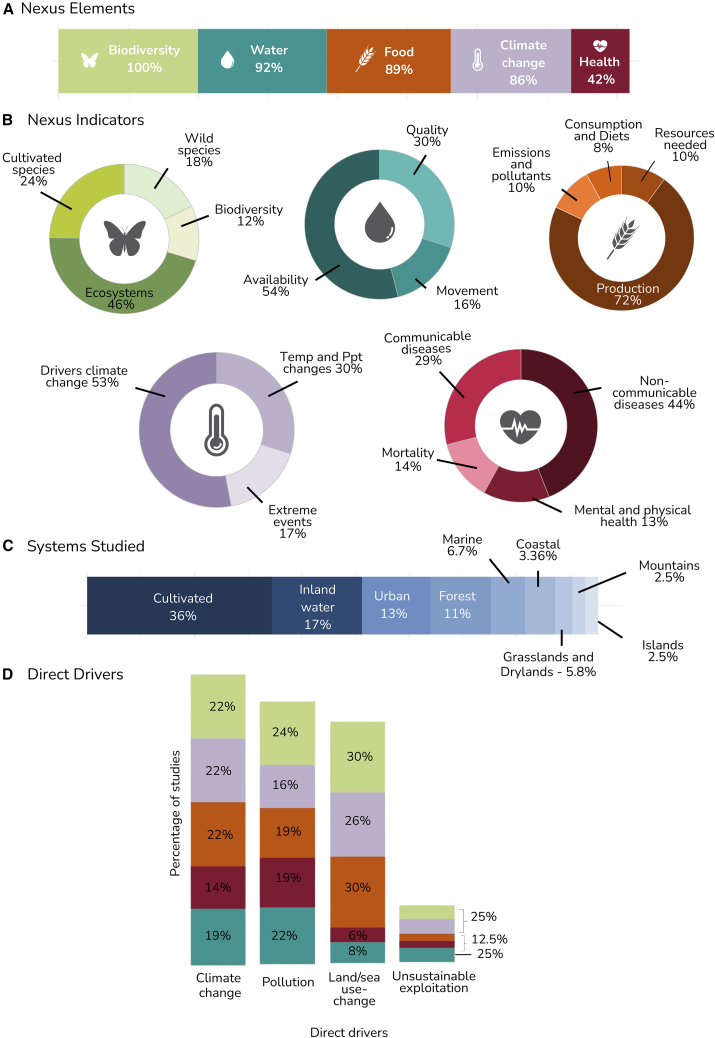
Summary of the biodiversity, water, food, health and climate elements and indicators found in the 84 studies evaluated (A) Frequency of each nexus element in the studies found; (B) frequency of each indicator used for each element; (C) frequency of each system studied using the nexus approach; and (D) percentage of studies evaluating each nexus element (indicated by the color) for each direct driver. Each nexus element is represented by a unique color in this figure: biodiversity is represented by light green; water is represented by teal; food is represented by orange; health is represented by dark red; and climate change is represented by light purple.

Water, food, and climate change were the most studied nexus elements, occurring in 78 (92.9%), 76 (90.5%), and 73 (86.9%) articles, respectively. Health was the most neglected element and included in only 36 (42.9%) multi-element articles (Figure 3A). Biodiversity was one of the systematic search inclusion criteria, thus by default was included in all articles. Most importantly, no single study evaluated the interactions of all five nexus elements as part of the analysis, indicating a significant gap.

The most frequent systems featured in the articles were cultivated (36.2%), inland water (17.8%), urban (13.4%), and forests (11.7%) (Figure 3C). “Ecosystem,” referring to the status, quality or composition of ecosystems, was the most prevalent category used for analysis of biodiversity (45.5%), followed by cultivated species (24.5%), wild species (18.0%), and general biodiversity (12.0%). Water availability was addressed in 54% of the articles and water quality in 30%. Production was the most addressed indicator for food, comprising 72% of all relationships analyzed compared to 10% for the indicator emissions and pollutants, 10% for the indicator resources needed, and 8% for the indicator consumption and diets (Figure 3B). Climate change and its indicators were addressed in 53% of the articles, with temperature and precipitation changes being present in 30%, and extreme events in 17% of articles (Figure 3B). Non-communicable diseases were addressed in 43.6% of articles; communicable diseases in 29.5%, human mortality in 14.1%, and mental and physical health in 12.7% (Figure 3B).

**Figure 4:**
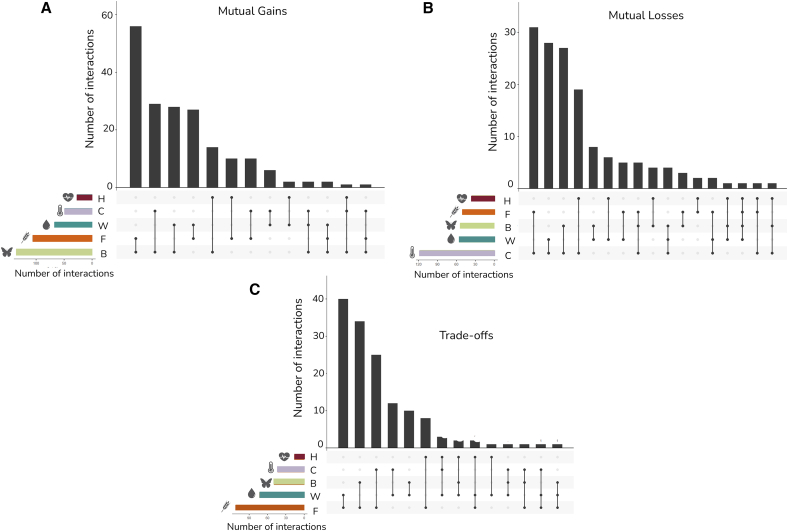
Numbers of interactions of mutual gains, mutual losses and trade-offs found for each combination of nexus element UpSet plots illustrate the number of interactions classified as (A) mutual gains, (B) mutual losses, and (C) trade-offs recorded for each combination of nexus element: biodiversity (B), food (F), water (W), climate change (C), and health (H). Each plot displays the number of interactions found (vertical black bars) for each nexus interaction combination containing two-way, three-way, or four-way interactions across nexus (cardinality) (black dots and lines indicating the elements involved). Filled circles in the matrix indicate which nexus elements are included in a given intersection and connected by lines to highlight shared interactions. Together, these plots highlight the degree of coherence or divergence in outcomes across the nexus elements, providing an integrated view of where synergies and trade-offs most frequently occur within the nexus. Each nexus element is represented by an icon: biodiversity (butterfly), water (droplet), food (grain), health (heart), climate change (thermometer).

In studies that evaluated interactions including food, the most frequently evaluated relationships involved aspects related to production (72.4%). Production relationships, i.e., food or agricultural commodity production (see Carsan et al. 2014^54^), are often connected to other aspects related to agricultural systems in the other nexus elements, such as cultivated species within the biodiversity system (39.02%), water availability in the water system (59.4%), and climate change and its drivers in the climate system (37.7%). In addition, temperature and precipitation changes were also frequently mentioned as a result of food production (37.7%). For the climate element, the most common relationship was between food and climate change (Figure 4), often exploring how producing food creates greenhouse gases, or the complicated relationship of changes in temperature and precipitation either supporting or harming crop growth, depending on the crop species and geographical region. In addition, shifting temperatures and precipitation against water availability was addressed in 45% of the articles on water-climate interactions.

For studies involving biodiversity, the interlinkages between ecosystem and water availability (23.2%), cultivated species and food production (28.6%), ecosystem and climate change (23.9%), and ecosystem and mental and physical health (26.1%) were the most common.

Water availability and food production was the most common relationship assessed in water studies, present in 23.8% of the interlinkages addressed; it was also significantly assessed in regard to climate change, i.e., temperature and precipitation changes (14.0%), and with relation to the integrity or degradation of ecosystems (23.7%), i.e., healthy ecosystems and flooding risk, for example. The relationship between water quality and food production was also mentioned (8.8%).

The health element was the most underdeveloped, being involved in only 64 (out of 477) distinct relationships. However, four distinct relationships were mostly addressed: (1) non-communicable diseases as the result of climate change and its drivers (10.3%) or through pollutants and emissions from production of food (10.3%); 2) communicable diseases and the link to temperature and precipitation changes (9.0%); 3) the risk to human mortality from flooding (5.1%) or other extreme climate events (5.1%); and (4) mental and physical health impacts from the state of ecosystems (7.7%).

### Indirect and direct drivers shaping the nexus

Direct drivers, i.e., land and sea use change, unsustainable exploitation of resources, pollution, invasive alien species and climate change, were mentioned in 29 (35%) studies, with only two studies addressing multiple drivers; indirect drivers were mentioned 36 times (42%). Land-/sea-use change (35%) and pollutants (31%) were the most prevalent direct drivers, while demographic changes were the most prominent indirect driver (44%). Climate change, pollution, and land and sea use change are the three direct drivers whose impacts were studied across all five nexus elements (Figure 3D).

Over 50% of interactions involving biodiversity and food were impacted by land- and sea-use change. Pollutants showed no dominant pattern, affecting all elements of the nexus, although 20% of the interactions involved health. For articles including climate change as a nexus element, only 20% identified climate change as a direct driver, in 34% of the interactions analyzed. Looking at indirect driver relationships, 46% of all interactions were influenced by demographic changes, with food assessed in 54% of these cases. In 59% of cases where technology was recorded as an indirect driver, food was also one of the elements involved. Governance drivers were reported in only 4% of the recorded interactions. Where economics was noted as a key indirect driver, 30% of the interactions involved health.

### Synergies and trade-offs between the nexus elements

A total of 477 interactions comprising 336 synergies (188 for mutual gains and 148 for mutual losses) and 141 trade-offs were recorded (Figure 4). Table S2 contains the number of interactions in all papers listed for each coded relationship. The only four-way interaction found in the literature assessed the effects of negative water quality on ecosystems and consumption and diets, and then those effects on non-communicable diseases.^55^ Three-way interactions were most likely to involve climate change as an entry point (42.0%), followed by biodiversity (19.4%), or both climate change and water (16.1%). Specifically, the cascading effects of negative climate change on the other nexus elements were the most present of the three-way interactions, with only one link exploring climate change mitigation as an entry point in a three-way interaction.

**Figure 5:**
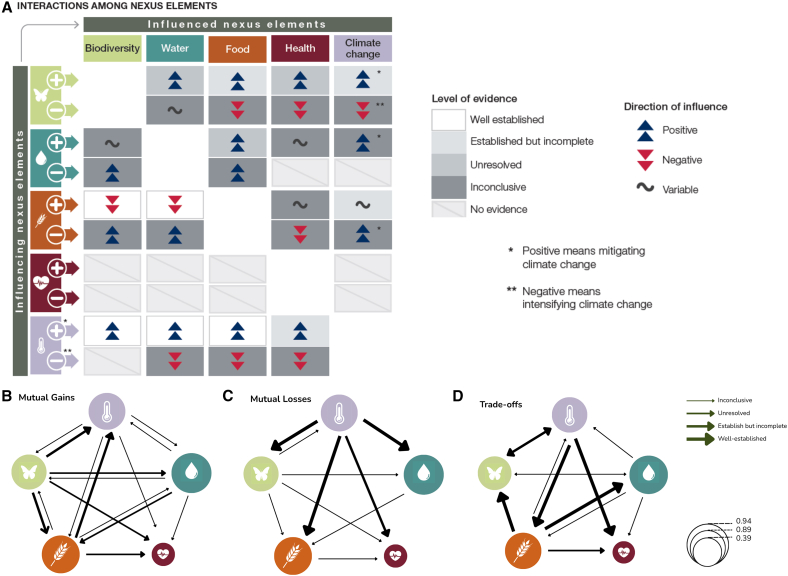
Resulting interactions among the different nexus elements (A) Matrix shows the evidence and directionality of interactions between the nexus elements based on the systematic literature review of studies including four and five nexus elements. The overall effect of either positive/improving (+) or negative/declining trends in a nexus element, denoted by the (+) or (−) sign in the left column, on other nexus elements is displayed, with the number of studies finding each effect being shown by the level of confidence. In this matrix, a positive effect on climate change means mitigation, while a negative effect means intensification. (B), (C) and (D) show the overall effect of the interactions classified as positive synergies, or mutual gains, negative synergies, or mutual losses, and trade-offs. The size of the dots represents the percentage of total studies incorporating each element, while the width of the arrows indicates the number of positive or negative effects found in the studies. Nexus element colors are the same as those used in Figure 3. For (B), (C), and (D), each nexus element is also represented by an icon: biodiversity (butterfly), water (droplet), food (grain), health (heart), climate change (thermometer).

There were four two-way interactions which consistently recorded a significantly higher number of positive synergies, or mutual gains: biodiversity on food (92.7% of all biodiversity-food records), biodiversity on water (81.8% of all biodiversity on water records), biodiversity on climate (85.3% of all biodiversity-climate record) and biodiversity on health (68.4% of all biodiversity-health records) (Figure 4; Figures 5A and 5B). This highlights the importance of biodiversity in maintaining the flow of ecosystem services that are essential for human health and well-being, including for climate change mitigation. Other important interactions that lead to mutual gains were water and food (72.4% of all water-food records) and food and health (52.6% of all food and health records), highlighting the importance of water for food provisioning and of food to human health.

Mutual losses in two-way interactions most frequently involved climate, with the worsening of climate change leading to negative effects on biodiversity (100% of all climate-biodiversity interactions), water (69.2% of all climate-water records), food (80.6% of all climate-food records) and health (100% of all climate and health records). This pattern highlights the dominant role of climate change as a driver that undermines the environmental support systems provided by biodiversity (Figures 5A and 5C). For example, there were 67 recorded interactions including biodiversity and climate, of which 47.7% resulted in positive synergies, when biodiversity acted as the entry point (meaning changes in biodiversity directly affecting climate), and 44.8% resulted in mutual losses, when climate acted as a direct driver. Trade-offs most frequently involved food, with increases in food leading to detrimental effects on biodiversity and water in 87.2% and 82.9% of the interactions recorded, respectively. Food and climate interactions presented compounding negative effects, with increasing food worsening climate change (66.7% of all food-climate records), and worsening climate change decreasing food (83.7% of all climate-food records).

## Discussion

The world is facing an unprecedented, interconnected polycrisis of biodiversity loss, water and food insecurity, human health threats and climate change. However, policies often address these crises in isolation, creating inefficiencies, conflicting goals and allowing harmful practices to persist. Improvements in our understanding of the complexity of interactions can potentially help contribute to more integrated policies, and this systematic review sought to address this knowledge gap by examining how trends in one element of the nexus influence others. It identified the key drivers shaping these interactions and highlighted which elements contribute most to positive impacts or further degradation across the nexus.

Most of the studies found relationships showing mutual gains between biodiversity and water, food, health and climate regulation, underscoring biodiversity’s fundamental role in supporting and sustaining these interconnected nexus elements. Similarly, mutual losses were observed between climate and biodiversity, water, food and health, underscoring the dominant role of climate change as a driver that weakens the environmental support structures sustained by biodiversity. Trade-offs were observed between food and the other elements, highlighting the role of food production as a driver shaping both positive and negative outcomes across the nexus elements. Notably, health was the least studied element despite previous research emphasizing its importance within a nexus context.^56^

### Nexus research on biodiversity, water, food, health, and climate change

Biodiversity was one of the criteria for inclusion in the systematic review and was therefore included in all articles. However, most articles considered biodiversity in its most general sense, such as the presence of green areas, types of land cover or ecosystems, or certain useful species mainly important for food production, corroborating what was found in Kim et al. (2024)^42^ in an analysis of nexus studies in the European context. Less than a quarter of the articles mentioned species and gene diversity, showing a lack of good indicators for biodiversity in studies applying a nexus approach. Biodiversity encompasses many interacting levels, from genetic to landscape diversity, each of which is important for the delivery of ecosystem services.^57^ For instance, studies have shown the positive effects of species diversity on ecosystem functioning,^58^ the stability and resilience of grassland and forest communities,^23^^,^^59^^,^^60^ which is important for the provision of services such as climate regulation, drinking water supply, soil erosion prevention, and forage production in natural grasslands. Species diversity also enhances the capacity of wetlands and aquatic ecosystems to purify water,^61^^,^^62^ while the diversity of soil biota underpins soil fertility, which is vital for food production.^63^^,^^64^^,^^65^ Nevertheless, there are still few studies that investigate the interactions among these levels and different aspects of biodiversity simultaneously.^26^ As our analysis shows, nexus-related studies most often conceptualize biodiversity either as whole ecosystems or at the species level, thereby neglecting its contributions to human well-being at other levels of biological organization.

Water, food, and climate change were included in most of the articles addressing multiple interactions, but with a low number of indicators used for each nexus element. Water availability, food production, and temperature and precipitation changes were the most common indicators assessed in the articles. This pattern reflects the dominance of certain sectors within the nexus literature, particularly food production, which is directly and critically dependent on water availability and which can act as a driver of climate change. Increases in food production, which have increased over the last 50 years,^51^ account for 80% of humanity’s total water demand, totaling 7,212 km3/year out of a total of 9,008 km3/year.^66^^,^^67^^,^^68^^,^^69^^,^^70^ Despite this increase, 800 million people remain undernourished (i.e., suffer from nutrient deficiency),^36^ while substantial quantities of food are lost each year.^71^^,^^72^ Furthermore, unsustainable agricultural practices have economic consequences, with agricultural losses from environmental degradation estimated to be valued at $231 billion per year (2007 values).^73^ These points, and the fact that food production is also important for human health outcomes,^51^ may explain the predominance of food production and water availability within nexus studies.

Climate change was present in almost half of the interactions recorded, showing the importance of this element as a driver within or being affected by the nexus. For example, in the Pre-Aral region of Central Europe and Asia, climate change impacts resulting in poor drinking water, aridity, and poor nutrition, combined with low incomes, have resulted in high infant and child mortality. As a result, the city of Muynak in Uzbekistan has undergone a 90% reduction in population over the course of the last few years as communities abandon the region.^74^ This shows how climate change effects can cascade within the nexus and interact with economic conditions, increasing the intensity of health impacts.

Health was the least developed element in nexus studies, being involved in only 64 distinct relationships. Despite being the most neglected element, the existing studies demonstrated strong interconnections between health and the other nexus elements, underscoring the importance of incorporating health into nexus research. For example, schistosomiasis is an acute and chronic tropical disease caused by a worm of the genus *Schistosoma,* which impairs the growth and nutrition of children and the physical work capacity of adults. Biodiversity loss due to increases in food production, industrialization, urbanization, and associated increased population mobility have influenced patterns of transmission and infection, with increased risks to agricultural production in rice fields infested with the intermediate snail host, and to rates of the infection in livestock.^75^ Risk of *Schistosoma japonicum* infection is also influenced by the domestic environment, including both the location of the house in relation to snail-colonized water sources, access to safe water, and improved sanitation. Household wealth and income determine family ability to provide and maintain safe water and sanitation, while also determining or interacting with other variables.^75^ Although climate change,^76^^,^^77^ biodiversity loss and urbanization^78^ are also known threat multipliers for many emerging infectious diseases, there remains a knowledge gap about the interactions of the various nexus elements and the emergence and spread of zoonotic pathogens.

### Indirect and direct drivers shaping the nexus

Land-/sea-use change was unsurprisingly the most frequently reported direct driver assessed in the articles using a nexus lens. Land-use change is a key driver of biodiversity loss^79^ and is directly linked to food production—on average, 90% of forest loss can be attributed to agriculture.^80^^,^^81^ Land-use change is also one of the main drivers of emerging infectious diseases and is responsible for 21% of global greenhouse gas emissions, thereby contributing to climate change.^82^ In addition, land-use changes that reduce evaporation, such as deforestation and wetland drainage, generally lead to increased runoff in watersheds locally and decreased precipitation downwind, affecting water availability.^27^^,^^28^^,^^83^^,^^84^^,^^85^

The impacts of land-use change within the nexus can be transboundary, i.e., deforestation in Amazonia and Central Africa affects precipitation in Europe, North America and Asia.^86^ Through teleconnections, the negative impacts of tropical deforestation on climate extend well beyond the tropics and limit food production in other regions, including the USA, India and China.^86^

The impacts of land-use change within the nexus also have economic consequences. For example, in La Via Lactea, Nicaragua, 93% of forest was converted for cattle ranching (pasture), which increased provisioning services but resulted in trade-offs, including losses of US$15.6m and US$4.4m in supporting and regulating ecosystem services, respectively, between 1978 and 2011.^87^ In Malawi, land-use and land cover changes projected between 1997 and 2035 could reduce ecosystem service values by 15% (from US$88.7m to US$76m.^88^). However, agroforestry systems may mitigate such trade-offs: Despite a 25% decline in productivity (but with high quality grains), soil carbon can increase by 36% and nitrogen leaching decrease by 50% in a 15-year period.^89^ These examples highlight how management intensity fundamentally shapes outcomes for ecosystem services.

Pollution, while less consistently reported across nexus interactions, has significant implications for both ecosystem and human health, with cascading effects across the other nexus elements. For example, total anthropogenic emissions of nitrous oxide, a potent greenhouse gas primarily produced from mineral fertilizer use and a precursor to tropospheric ozone, have increased by about 30% from 1980 to 2016.^90^ As a consequence of increased ozone exposure, crop yields, such as maize, rice, soybean, and wheat, have reduced by approximately 3–7% in the early 21st century.^91^

Globally, 64% of agricultural land is at risk from pesticide pollution, with runoff impacting major river basins, particularly in South Africa, China, India, Australia, and Argentina.^92^ Excessive nutrient loads in food production have also contributed to freshwater and marine biodiversity loss, with many river estuaries and coastal zones being characterized by dead zones. Currently, more than 400 dead zones in coastal waters around the world have been identified, affecting a total area of more than 245,000 km^2^ (about the size of the United Kingdom), where excess nutrients lead to areas of low to no oxygen that can kill fish and other marine life.^93^^,^^94^

Pollution also affects human health, being responsible for 9 million premature deaths annually, with air pollution causing 6.7 million and water pollution 1.4 million deaths.^95^ More than 80% of industrial and municipal sewage from human activities discharged into rivers, estuaries and oceans are untreated,^96^ and consequently more than 50 diseases are caused by poor water supply, water quality and sanitation.^97^ Poor water quality and related activities (including drinking water, sanitation and hygiene) have been conservatively estimated to result annually in 1.4 million deaths and 74 million DALY (disability-adjusted life year), with a high burden in the poorest countries.^95^^,^^98^

Climate change was analyzed as a direct driver in almost 34% of the interactions assessed and in 15% as an element being affected by another nexus element. When analyzed as a driver, food, water and biodiversity were the most common elements, with the effects of climate change on biodiversity being well studied. For example, ocean acidification has negatively impacted coral reefs ecosystems and subsequently the health and well-being of people whose livelihoods depend on these systems.^99^^,^^100^ Altered hydrological cycles in mountain catchments due to climate change have reduced water discharge, affecting millions of people in large catchments, even far away from mountain areas.^101^^,^^102^^,^^103^^,^^104^^,^^105^ In the past two decades, this caused severe water crises for 60–70% of the Himalayan population that directly rely on springs for drinking water and irrigation.^106^^,^^107^ Jointly with warmer temperatures, the altered runoff also has a negative impact on food production^102^ (e.g., due to less water being available for irrigation), affecting food security and human well-being. Climate change effects on human health are also well established: 74 temperature-related excess deaths per 100,000 inhabitants globally occurred between 2000 and 2019.^108^ In the past 50 years, extreme weather, climate and water-related events have caused nearly 12,000 disasters, with 2 million human deaths (90% of which are in low-and-middle-income countries) and $4.3 trillion in total costs globally.^109^

### Synergies and trade-offs between the nexus elements

Overall, the systematic review revealed consistent relationships between biodiversity, food, water, health and climate change. The role of biodiversity in improving the other nexus elements is well supported by the evidence - increases in biodiversity can increase food production and quality, diversify diets, improve water quantity and quality, improve human health and mitigate climate change. For example, species, trait and genetic diversity in crops, their wild relatives and other harvested wild species are key to food diversity and its production.^110^ Forests provide 75% of the world’s accessible freshwater for agricultural, domestic, industrial and environmental uses,^36^ and an increase in 10% of forest cover in a watershed can reduce water treatment costs by an average of 5%.^111^^,^^112^ Examples of synergies between biodiversity and health include the presence and number of green spaces (which includes biodiversity) positively influencing mental and physical health,^113^^,^^114^^,^^115^ wetland biodiversity positively impacting clean air and subsequently human health and increased freshwater biodiversity leading to decreases in waterborne disease.^115^

It is often difficult to determine the causal direction of synergies and trade-offs as the nexus interactions are complex, and different studies show different outcomes. However, some relationships can be generalized: When there is a loss of biodiversity, negative cascading effects on the other nexus elements are observed (i.e., mutual losses; Figures 5B and 5C), corroborating what was found in nexus studies in Europe which included biodiversity, climate, food, water, energy, transport and health.^42^ In India, the loss of pollinator biodiversity has led to a decline in soil quality, which combined with poor water management and climate change has led to a loss of agricultural production.^116^ In the Brazilian Amazon, 70% of deforested land has been converted to cattle ranching, resulting in biodiversity loss, decreases in water availability and quality, and contributing to climate change. However, these impacts are non-linear, and, under some conditions, deforestation can lead to increases in soil carbon.^117^

Well-established trade-offs were observed between food and the other elements, with increases in food production being consistently linked to biodiversity loss, reduced water quantity and quality, and worsening climate change.^118^^,^^119^^,^^120^ The relationship between food production and health, however, remains inconclusive: while increases in food quantity can improve some aspects of human health, insufficient access to nutritious food (i.e., decreases in food quality) can be detrimental (Figures 5A–5C). Water usage due to increased food production, fishing, and industry has complex impacts on the climate at larger scales: Organic sheep farming in Finland increased the genetic diversity of the biosphere and biogeochemical flows (synergies), but concurrently caused trade-offs and negative impacts on climate, freshwater use, and crop production.^121^ However, if agri-environmental practices are implemented, food production can have synergistic and positive effects on all other elements^122^: the maintenance and/or development of both grasslands and new ponds concurrently together enhance water quality, increase carbon sequestration, lower emissions, increase biodiversity, and provide cultural (health and well-being) services.^123^ In California, applying multiple regenerative practices concurrently in the production of almonds, including composts, reducing agrichemical inputs, and putting aside land for non-crop habitat, increased biodiversity and has resulted in a doubling of profits for farms using these practices.^124^ A study in India estimated that restoring degraded soils and changing agricultural practices could increase the total potential of soil carbon sequestration by 44 ± 5 Tg C/y,^125^^,^^126^^,^^127^ demonstrating how shifts in food production practices can contribute to the mitigation of climate change.

### Limitations of the study

This systematic review has several limitations that should be acknowledged. First, the review looked for 4- or 5-way interactions between elements of the nexus and thus does not provide a complete overview of less complex relationships involving two and/or three elements. While the sampling design was intended to capture more complex relationships, studies mentioning four and five interactions were still only evaluating less-complex relationships between two or three elements, a similar result to a recent regional analysis.^42^ There are several possible reasons for this: Firstly, studying all five nexus elements concurrently is complicated and difficult to undertake and, therefore, is likely to be rarely achieved. For example, Zheng et al. (2019),^128^ evaluated biophysical strategies to achieve simultaneous gains in production, ecosystem services, biodiversity, and local livelihoods through integrated and diverse interdisciplinary methods. Secondly, interdisciplinary science is complex and requires specific methodologies, incentives, and training for its realization, as well as a high level of collaboration between different disciplines.^129^ As a result, there is still a lack of funding and methodological approaches to allow for more interdisciplinary research. Methods are often applied in isolation or combined only with traditional approaches, rather than being integrated into broader, more innovative methodological frameworks, limiting research methods grounded in complexity science.^130^ Today’s science system acknowledges and supports specialization rather than holistic, interdisciplinary thinking,^131^ which can be a reason for the lack of studies that employ a nexus lens.

Other interdisciplinary frameworks, such as One Health and Planetary Health, also exist, aiming to facilitate collaboration, communication, capacity building, and coordination across sectors to tackle these problems.^129^ However, so far, they also have limited appearances in the literature involving more than three elements. Thirdly, this systematic review focuses on five elements: biodiversity, water, food, health, and climate change (with the mandatory inclusion of biodiversity in the articles), and does not include other elements such as energy. These factors may have limited the number of articles found, since the WEF nexus, for example, generally dominates the literature found.^46^ In addition, the systematic review only considered studies published in English (i.e., the common language of science), potentially omitting a variety of studies that address more complex nexus element interactions. It is important to note that this study did not evaluate the methodological quality or scientific impact of the analyzed papers, such as sampling design, analytical robustness, or other aspects of methodological rigor, which should therefore be considered a limitation. Nevertheless, to reduce potential bias and ensure a minimum quality standard, the search was restricted to articles published in peer-reviewed and indexed journals.

### Conclusions and implications

The nexus approach reveals the complexity and interdependence of biodiversity, water, food, health and climate change in addressing current environmental challenges. Despite the urgent need to tackle these interlinked global crises, our results show that interactions involving more than three nexus elements remain poorly studied. Nevertheless, the evidence is clear: Biodiversity plays a fundamental role in the nexus, driving positive interactions and providing essential support systems for the robust and healthy ecosystems that sustain both people and the planet. Conversely, food production and climate change are major drivers of biodiversity loss, water stress, and adverse health outcomes. Food production also contributes to climate change while simultaneously being impacted by it, exemplifying the complex feedbacks within the nexus.

Building on the results of our analysis, there are strong policy implications. Policies that conserve biodiversity and mitigate climate change can generate far-reaching benefits across all nexus elements, given biodiversity's importance as an underpinning element and climate as a negative driver. Supporting long-term sustainable resource management and advancing global sustainability goals will require integration with sustainable food production strategies to achieve even greater potential to deliver outcomes across the entire nexus. However, many current policies are often developed in isolation within institutional or sectoral silos, leading to trade-offs and unintended consequences.^47^ In addition, policies need to be tailored to local contexts, since some of these relationships can be context-dependent and vary across social-ecological systems, reflecting differences in land use history, governance, and the degree of human modification of landscapes. The nexus approach offers a solution: By integrating data and knowledge across all elements, we can maximize synergies, minimize trade-offs, reduce costs, and ensure a sustainable future.^132^

## Acknowledgments

We thank the members of the IPBES Bureau and Multidisciplinary Expert Panel, including those who were members of the Nexus Assessment management committee and provided guidance throughout the assessment process. We also thank the external reviewers who provided thoughtful comments on drafts of Chapter 2 during the production of the Nexus Assessment. The leadership, guidance, and support of the IPBES secretariat have been invaluable throughout the assessment process. Authors gratefully thank contributions received from funders that are different from authors' affiliations: DTSH is supported by the Percival Carmine Chair in Epidemiology & Public Health (RM#25412); 10.13039/100019980GBS by the collaborative project under the university corporate collaboration agreement between Kubota Corporation and The 10.13039/501100004721University of Tokyo. D.O. obtained support from 10.13039/100007843NORAD (RAF-3058 KEN-18/0004); E.M. from the 10.13039/501100000275Leverhulme Trust, United Kingdom (Early Career Fellowship); F.G. from the 10.13039/501100018818National Research, Development and Innovation Office, Hungary (RRF-2.3.1-21-2022-00006); L.B.F from the German Federal Ministry for Research, Technology and Space (BMFTR), Germany (LANUSYNCON, 01UU2002).

## Author contributions

P.R.P., A.B., E.B., E.M., and C.H. were responsible for data curation and formal analysis. P.R.P., A.B., and C.H. were responsible for writing the original draft. All authors contributed to the conceptualization and writing – review and editing.

## Declaration of interests

The authors declare no competing interests.

## Supplemental information

Document S1. Table S1 and S2

Data S1. SOM-data coding
