# Supplementary material for: Beyond the silos: A global evidence synthesis of biodiversity, water, food, health and climate nexus interactions
Source: iScience. 2026 Jul 22;29(8):116871. doi: 10.1016/j.isci.2026.116871 (PMC13426218; doi:10.1016/j.isci.2026.116871)
Supplement: Document S1. Table S1 and S2 [file mmc1.pdf]

iScience, Volume *29*

Supplemental information

Beyond the silos: A global evidence synthesis of biodiversity, water, food, health and climate nexus interactions

Paula R. Prist, Allison Bailey, Elena Bukvareva, Eva Maire, Ralf Seppelt, David T.S. Hayman, Lisa Biber-Freudenberger, Sunita Chaudhary, Pradeep Kumar Dubey, Ronald C. Estoque, Yuka Estrada, Gábor Földvári, Paula A. Harrison, Abid Hussain, Tiff van Huysen, Roxanne Suzette Lorilla, Silvia Francis Materu, Pamela McElwee, Ernest L. Molua, David Obura, Giles B. Sioen, and Caroline Howe

Supplementary Material

Table S1. Keyword combination used in Scopus and the number of articles returned. The search was performed on 21 July 2022. The first row shows the naive keywords used in the *litsearchr* package. Other rows show the keywords from *litsearchr* package for all Nexus, four and three-order interactions. The keyword combination used spans between the rows 1 to 7.

	**Keywords**	**No. of articles**	**Nexus type**
1	(Biodiversity OR diversity* OR landscape* OR ecosystem) AND (Food OR food* OR food security OR productivity OR nutrition) AND (Water OR water* OR hydro*) AND (Climate OR Climate change OR Climate*) AND (Human health OR well-being OR human* OR zoono*)	686	Complete - naïve search
2	("ecosystem service" OR "natural resource" OR "sustainable development" OR "environmental sustainability" OR "natural ecosystem" OR biodiversity) AND ("human health" OR "human well-being" OR zoonotic OR "mental Health") AND ("water management" OR "water quality" OR "water security" OR "water use" OR "water availability" OR "water resource" OR "water supply") AND ("crop yield" OR "food production" OR "food security" OR "agricultural production" OR "production system" OR nutrition) AND ("carbon sequestration" OR "climate change" OR "greenhouse gas") AND ("environmental change" OR "environmental impact" OR "human activities" OR "land degradation" OR "land management" OR "land use" OR "economic development" OR "human population" OR "population growth")	53	Biodiversity – health-water-food-climate (with drivers)
3	("ecosystem service" OR "natural resource" OR "sustainable development" OR "environmental sustainability" OR "natural ecosystem" OR biodiversity) AND ("human health" OR "human well-being" OR zoonotic OR "mental Health") AND ("water management" OR "water quality" OR "water security" OR "water use" OR "water availability" OR "water resource" OR "water supply") AND ("crop yield" OR "food production" OR "food security" OR "agricultural production" OR "production system" OR nutrition) AND ("carbon sequestration" OR "climate change" OR "greenhouse gas")	108	Biodiversity – health-water-food-climate (without drivers)
4	("ecosystem service" OR "natural resource" OR "sustainable development" OR "environmental sustainability" OR "natural ecosystem" OR biodiversity) AND ("human health" OR "human	211	Biodiversity – health – water - food

	well-being" OR zoonotic OR "mental Health") AND ("water management" OR "water quality" OR "water security" OR "water use" OR "water availability" OR "water resource" OR "water supply") AND ("crop yield" OR "food production" OR "food security" OR "agricultural production" OR "production system" OR nutrition)		
5	("ecosystem service" OR "natural resource" OR "sustainable development" OR "environmental sustainability" OR "natural ecosystem" OR biodiversity) AND ("human health" OR "human well-being" OR zoonotic OR "mental Health") AND ("crop yield" OR "food production" OR "food security" OR "agricultural production" OR "production system" OR nutrition) AND ("carbon sequestration" OR "climate change" OR "greenhouse gas")	401	Biodiversity – health – food – climate
6	("ecosystem service" OR "natural resource" OR "sustainable development" OR "environmental sustainability" OR "natural ecosystem" OR biodiversity) AND ("human health" OR "human well-being" OR zoonotic OR "mental Health") AND ("water management" OR "water quality" OR "water security" OR "water use" OR "water availability" OR "water resource" OR "water supply") AND ("carbon sequestration" OR "climate change" OR "greenhouse gas")	365	Biodiversity – health – water - climate
7	("ecosystem service" OR "natural resource" OR "sustainable development" OR "environmental sustainability" OR "natural ecosystem" OR biodiversity) AND ("water management" OR "water quality" OR "water security" OR "water use" OR "water availability" OR "water resource" OR "water supply") AND ("crop yield" OR "food production" OR "food security" OR "agricultural production" OR "production system" OR nutrition) AND ("carbon sequestration" OR "climate change" OR "greenhouse gas")	1251	Biodiversity – water – food - climate
8	("ecosystem service" OR "natural resource" OR "sustainable development" OR "environmental sustainability" OR "natural ecosystem" OR biodiversity) AND ("human health" OR "human well-being" OR zoonotic OR "mental Health") AND ("water management" OR "water quality" OR "water security" OR "water use" OR "water availability" OR "water resource" OR "water supply")	1375	Biodiversity-health-water
9	("ecosystem service" OR "natural resource" OR "sustainable development" OR "environmental sustainability" OR "natural ecosystem" OR	1236	Biodiversity – health- food

	biodiversity) AND ("human health" OR "human well-being" OR zoonotic OR "mental Health") AND ("crop yield" OR "food production" OR "food security" OR "agricultural production" OR "production system" OR nutrition)		
10	("ecosystem service" OR "natural resource" OR "sustainable development" OR "environmental sustainability" OR "natural ecosystem" OR biodiversity) AND ("human health" OR "human well-being" OR zoonotic OR "mental Health") AND ("carbon sequestration" OR "climate change" OR "greenhouse gas")	2122	Biodiversity – health - climate
11	("ecosystem service" OR "natural resource" OR "sustainable development" OR "environmental sustainability" OR "natural ecosystem" OR biodiversity) AND ("water management" OR "water quality" OR "water security" OR "water use" OR "water availability" OR "water resource" OR "water supply") AND ("crop yield" OR "food production" OR "food security" OR "agricultural production" OR "production system" OR nutrition)	3898	Biodiversity – food – water
12	("ecosystem service" OR "natural resource" OR "sustainable development" OR "environmental sustainability" OR "natural ecosystem" OR biodiversity) AND ("crop yield" OR "food production" OR "food security" OR "agricultural production" OR "production system" OR nutrition) AND ("carbon sequestration" OR "climate change" OR "greenhouse gas")	5945	Biodiversity – food – climate
13	("ecosystem service" OR "natural resource" OR "sustainable development" OR "environmental sustainability" OR "natural ecosystem" OR biodiversity) AND ("water management" OR "water quality" OR "water security" OR "water use" OR "water availability" OR "water resource" OR "water supply") AND ("carbon sequestration" OR "climate change" OR "greenhouse gas")	7865	Biodiversity – water - climate

Table S2. Number of interactions in all articles that found positive (+) or negative (-) trend effects of the elements of the entry point of the nexus positively (+) or negatively (-) affecting the other elements in bidirectional relationships. Positive trends in climate were coded as climate change mitigation; negative trends in climate as climate change worsening. No interactions or articles were found using health as an entry point. Capital letters represent the five nexus elements: B: biodiversity, C: climate change, F: food, H: health and W: Water.

**Two-Way Interactions**				
**Entry point**	**Positive trends of a Nexus on another Nexus**	**Number of interactions**	**Negative trends of a Nexus on another Nexus**	**Number of interactions**
Biodiversity	+B to +B	0	-B to +B	0
	+B to -B	0	-B to -B	0
	+B to +W	20	-B to +W	2
	+B to -W	3	-B to -W	1
	+B to +F	53	-B to +F	0
	+B to -F	2	-B to -F	5
	+B to +H	14	-B to +H	0
	+B to -H	1	-B to -H	8
	+B to +C	32	-B to +C	0
	+B to -C	0	-B to -C	5
Water	+W to +W	0	-W to +W	0
	+W to -W	0	-W to -W	0
	+W to +B	12	-W to +B	1
	+W to -B	5	-W to -B	10
	+W to +F	22	-W to +F	2
	+W to -F	3	-W to -F	5
	+W to +H	2	-W to +H	0
	+W to -H	4	-W to -H	7
	+W to +C	4	-W to +C	2
	+W to -C	1	-W to -C	1
Food	+F to +F	0	-F to +F	0
	+F to -F	0	-F to -F	0
	+F to +B	4	-F to +B	1
	+F to -B	35	-F to -B	0
	+F to +W	6	-F to +W	1
	+F to -W	35	-F to -W	0
	+F to +H	10	-F to +H	0
	+F to -H	19	-F to -H	2
	+F to +C	9	-F to +C	0
	+F to -C	20	-F to -C	1
Climate	+C to +C	0	-C to +C	0
	+C to -C	0	-C to -C	0
	+C to +B	1	-C to +B	0
	+C to -B	0	-C to -B	30

	+C to +W	3	-C to +W	11
	+C to -W	0	-C to -W	34
	+C to +F	2	-C to +F	5
	+C to -F	0	-C to -F	39
	+C to +H	0	-C to +H	0
	+C to -H	0	-C to -H	26

**Three-Way Interactions**		
**Entry Point**	**Trend of a Nexus on another**	**Number of interactions**
Biodiversity	+B to +C & +H	1
	+B to +C & +H	1
	+B to +F & +W	1
	+B to +C & +F	1
	-B to -H & +W	2
Biodiversity & Climate	-B & -C to -F	3
Water	+W to +B & +F	1
	-W to -B & -F	1
Food	+F to -H & -W	1
Food & Water	+F & +W to -B	1
Climate	+C to +B & +W	1
	-C to -B & -F	2
	-C to -B & -H	1
	-C to -B & -W	4
	-C to -F & -H	1
	-C to -F & -W	3
	-C to -H & +W	1
Climate & Water	-C & +W to -B	1
	-C & +W to -F	1
	-C & +W to -H	3
**4-Way Interactions**		
**Entry Point**	**Trend of Nexus on another**	**Number of interactions**
Water	+W on -B & -F on -H	1
